# Association of CALLY index with NAFLD in U.S. adults from NHANES 2017–2020 assessed by vibration-controlled transient elastography

**DOI:** 10.1186/s13098-025-01926-y

**Published:** 2025-08-29

**Authors:** Xunge Lin, Xiaozhen Huang, Ziqun Yi, Minran Li, Xujing Liang

**Affiliations:** Department of Infectious Disease, the First Affiliated Hospital of Jinan University, Guangzhou, 510630 China

**Keywords:** CALLY index,, NAFLD,, Inflammation, NHANES, cross-sectional study

## Abstract

**Background:**

Non‑alcoholic fatty liver disease (NAFLD) is characterized by excessive hepatic fat accumulation and is closely associated with inflammation and metabolic dysregulation. The C‑reactive protein–albumin–lymphocyte (CALLY) index, a composite marker of inflammation, immunity, and nutritional status, remains understudied in relation to NAFLD.

**Methods:**

A crosssectional analysis was conducted using data from 7,271 U.S. adults in NHANES 2017–2020. NAFLD was defined by vibrationcontrolled transient elastography with a controlled attenuation parameter (CAP) > 274 dB/m. Weighted logistic regression, restricted cubic spline (RCS) modeling, and twopiecewise logistic regression were applied to assess linear and nonlinear associations between the CALLY index and NAFLD prevalence. Subgroup and sensitivity analyses were performed to evaluate the consistency and robustness of the findings.

**Results:**

The mean CALLY index was 8.08 (SD 12.42). Higher CALLY levels were inversely associated with NAFLD prevalence ( OR  = 0.96; 95% CI, 0.95–0.98). Compared with the lowest quartile (Q1 < 1.90), the highest quartile (Q4 > 10.00) showed a 61% lower prevalence of NAFLD (OR = 0.39; 95% CI, 0.24–0.64). RCS analysis demonstrated a significant nonlinear relationship, with a threshold at 8.91; below this value, each unit increase in the CALLY index corresponded to a 10% reduction in NAFLD prevalence (OR = 0.90; 95% CI, 0.88–0.92). Subgroup and sensitivity analyses yielded consistent results, confirming the robustness of these findings.

**Conclusion:**

The CALLY index demonstrates a significant inverse association with NAFLD prevalence and may serve as a simple composite indicator for identifying individuals at higher likelihood of NAFLD, providing additional insights to inform future screening and risk‑stratification research.

**Supplementary Information:**

The online version contains supplementary material available at 10.1186/s13098-025-01926-y.

## Background

Non-alcoholic fatty liver disease (NAFLD) is a common hepatic ailment characterized by excessive fat accumulation in hepatocytes, often associated with metabolic disorders such as metabolic syndrome, obesity, type 2 diabetes, and insulin resistance [1–4]. According to epidemiological research, the prevalence of NAFLD has risen to over 25% globally, and its prevalence is positively connected with growing rates of obesity and metabolic syndrome, especially in groups at high risk where the disease is more prevalent [5, 6]. Importantly, NAFLD is not a single pathological entity but represents a heterogeneous spectrum ranging from bland hepatic steatosis—defined by simple fat accumulation with minimal inflammation—to non-alcoholic steatohepatitis (NASH), which is characterized by hepatocyte ballooning and multicellular inflammatory responses that may progress to fibrosis, cirrhosis, and hepatocellular carcinoma [7, 8]. This pathological distinction has substantial clinical relevance, as recent studies, including a comprehensive review, highlight the critical role of genetic predisposition in modulating NAFLD susceptibility and progression, and its influence on therapeutic endpoints in emerging gene knockdown–based clinical trials [9]. Early symptoms of NAFLD are often non-specific, making clinical screening and early diagnosis challenging. Currently, liver biopsy remains the “gold standard” for diagnosing NAFLD, providing precise information on the extent of hepatic fat buildup and related inflammation or fibrosis [10, 11]. However, the procedure’s invasive nature, high cost, and associated risks limit its use in large-scale screenings [12, 13]. To address this, non-invasive imaging techniques like ultrasound and vibration-controlled transient elastography (VCTE) have been adopted for screening and evaluating NAFLD [7, 14, 15]. While these techniques enable population-level assessment of hepatic fat content and disease burden, their relatively high costs, operational complexity, and restricted accessibility impede widespread use. Consequently, identifying affordable, non-invasive, and readily accessible serological markers related to NAFLD could provide substantial value for screening high-risk populations and advancing large-scale epidemiological investigations.

Recognizing this pathological and genetic heterogeneity underscores the need to identify systemic biomarkers that capture metabolic and inflammatory status in relation to NAFLD at the population level. In this context, C‑reactive protein–albumin–lymphocyte (CALLY) index, an integrated biomarker reflecting inflammation, immune function, and nutritional status, may provide useful information for exploring early screening approaches and assessing the likelihood of NAFLD. Each component of the CALLY index is biologically relevant to NAFLD pathogenesis: albumin reflects hepatic synthetic function and nutritional reserve and is typically reduced in chronic inflammation [16]; lymphocytes contribute to immune surveillance and inflammatory regulation, and their decrease signals impaired immune homeostasis [17]; C-reactive protein (CRP) is an acutephase reactant that indicates systemic lowgrade inflammation and has been strongly associated with hepatic fat accumulation and progression to steatohepatitis [18]. It incorporates three serum markers: albumin, lymphocyte count, and CRP, which indicate nutritional, immunological, and inflammatory conditions. Previous research has demonstrated its significant predictive value in various chronic conditions. In individuals with cardiovascular illness, an elevated CALLY index is associated with improved survival rates and may offer protection against atherosclerosis and cardiovascular incidents [19, 20]. The CALLY index is associated with the advancement of metabolic syndrome and sarcopenia, where diminished levels signify greater metabolic abnormalities, low-grade inflammation, and malnutrition, correlating with elevated complication rates and mortality [21–23]. In oncology, the CALLY index has been used to assess systemic inflammation, immune status, and cancer prognosis, showing predictive value in patients with gastric, liver, lung, and other cancers [24–26]. Although the predictive efficacy of the CALLY index in metabolic disorders, cardiovascular illnesses, and cancers is well-documented, its utilization in liver diseases, especially for screening and forecasting NAFLD progression, is comparatively under-researched. Studies demonstrate that chronic low-grade inflammation is crucial in the advancement of NAFLD [27], and the CALLY index, which reflects inflammation, nutrition, and immune status, may provide insights into its association with NAFLD and inform future research on potential risk stratification. However, investigations on the association between the CALLY index and NAFLD remain scarce, and there is no conclusive epidemiological evidence endorsing its application as a risk assessment instrument for NAFLD.

This study examines the relationship between the CALLY index and NAFLD using data from the 2017–2020 NHANES database, evaluating its suitability as a biomarker for NAFLD. We propose an inverse correlation between the CALLY index and NAFLD, indicating that reduced CALLY index values may imply an increased probability of NAFLD. This work aims to explore the potential of a novel biomarker for identifying individuals with a higher likelihood of non-alcoholic fatty liver disease and to provide preliminary evidence that may inform future studies on risk stratification and management strategies.

## Methods

### Study population

This study conducted a cross-sectional analysis of NHANES data to evaluate the health and nutritional status of the U.S. population. NHANES employs a multistage, stratified sampling technique to guarantee national representativeness. Data collection is conducted every two years and includes questionnaires, physical exams, and laboratory tests, addressing disease status, nutritional indicators, lifestyle factors, and socioeconomic data. All participants granted informed consent for this study authorized by the NCHS Institutional Review Board. The study utilized NHANES data from 2017 to 2020, initially including 15,560 participants. Participants were excluded for the following reasons: (1) age < 20 years; (2) absence of VCTE data, specifically controlled attenuation parameter (CAP) values required to define NAFLD; (3) absence of laboratory parameters required to calculate the CALLY index, including serum albumin, lymphocyte count, or CRP.

### Calculation of the CALLY index

The exposure variable in this study is the CALLY index, which was calculated using the formula from previous research [28–30]:

$$\:\text{C}ALLY=\text{A}\text{l}\text{b}\text{u}\text{m}\text{i}\text{n}\text{*}\frac{\text{l}\text{y}\text{m}\text{p}\text{h}\text{o}\text{c}\text{y}\text{t}\text{e}}{C-reactive\:protein*10}$$

Where albumin is measured in g/dL, lymphocyte count in 10⁹/L, and CRP in mg/dL. In this study, laboratory data provided by NHANES included concentrations of albumin, lymphocyte count, and CRP. All these data were obtained from certified laboratories using standardized biochemical testing procedures to ensure data accuracy and consistency.

### Definition of non-alcoholic fatty liver disease

In this study, the outcome variable assessed is NAFLD, measured quantitatively using VCTE. VCTE utilizes transient shear wave elastography , a non-invasive, quantitative method to evaluate hepatic steatosis, commonly used in both epidemiological research and clinical screenings. The apparatus analyzes ultrasonic attenuation linked to fat deposition and documents the CAP, which assesses the degree of hepatic fat. This study established a median CAP value of > 274 dB/m as the diagnostic threshold, a criterion validated in extensive population investigations and demonstrated adequate diagnostic accuracy in detecting NAFLD [31–33]. Due to the lack of histological data in the NHANES database, simple steatosis cannot be directly distinguished from NASH. Therefore, hepatic steatosis assessed by VCTE was used as a surrogate indicator for NAFLD in this study. This definition has high feasibility in largescale epidemiological studies and can reasonably reflect the populationlevel prevalence characteristics of NAFLD.

### Covariate selection

To minimize potential confounders and improve analysis accuracy, this study incorporated a variety of sociodemographic, lifestyle, and health-related factors, including age, sex, race, body mass index (BMI), education, smoking status, physical activity, diabetes, and coronary artery disease (CAD). BMI was categorized utilizing NHANES data as underweight (< 18.5 kg/m²), normal weight (18.5–24.9 kg/m²), and obese (≥ 25 kg/m²) to assess the impact of various weight statuses on NAFLD [34]. Smoking status was categorized based on NHANES data, with individuals smoking ≥ 100 cigarettes in their lifetime classified as smokers, and the remainder as non-smokers [35]. Physical activity levels were categorized by metabolic equivalents (MET), with MET < 600 deemed inactive and ≥ 600 classified as active, to evaluate the influence of physical activity on metabolic health [36]. Diabetes and CHD diagnoses were based on self-reported medical history, including whether the participant had been diagnosed by a doctor. The study aims to incorporate these factors to better account for confounders, reduce bias, and improve the reliability of the analysis on the relationship between the CALLY index and NAFLD, hence ensuring the robustness and relevance of the findings.

### Statistical analysis

This study analyzed NHANES data from 2017 to 2020, incorporating appropriate sampling weights, strata, and primary sampling units to ensure nationally representative estimates. Continuous variables were summarized as weighted means with standard errors, and categorical variables as weighted percentages. To investigate the relationship between the CALLY index and NAFLD, weighted univariate and multivariate logistic regression analyses were performed. The CALLY index was analyzed both as a continuous variable and in quartiles (Q1: <1.90; Q2: 1.90–4.39; Q3: 4.39–10.00; Q4: >10.00) to evaluate doseresponse patterns. Restricted cubic spline (RCS) models were employed to explore potential nonlinear relationships; when nonlinearity was detected, twopiecewise logistic regression was performed to estimate odds ratios (OR) on either side of the identified inflection point. Subgroup analyses were conducted to assess potential effect modifiers across demographic and lifestyle strata, with interaction terms tested in the models. Sensitivity analyses were performed to examine the robustness of findings, including exclusion of participants with CALLY index values beyond the mean ± 3 SD and exclusion of individuals with a history of tumor. All statistical analyses were performed using R (version 4.2.1), with a p-value < 0.05 indicating significance.

## Results

### Participant selection and baseline characteristics

**Fig. 1:**
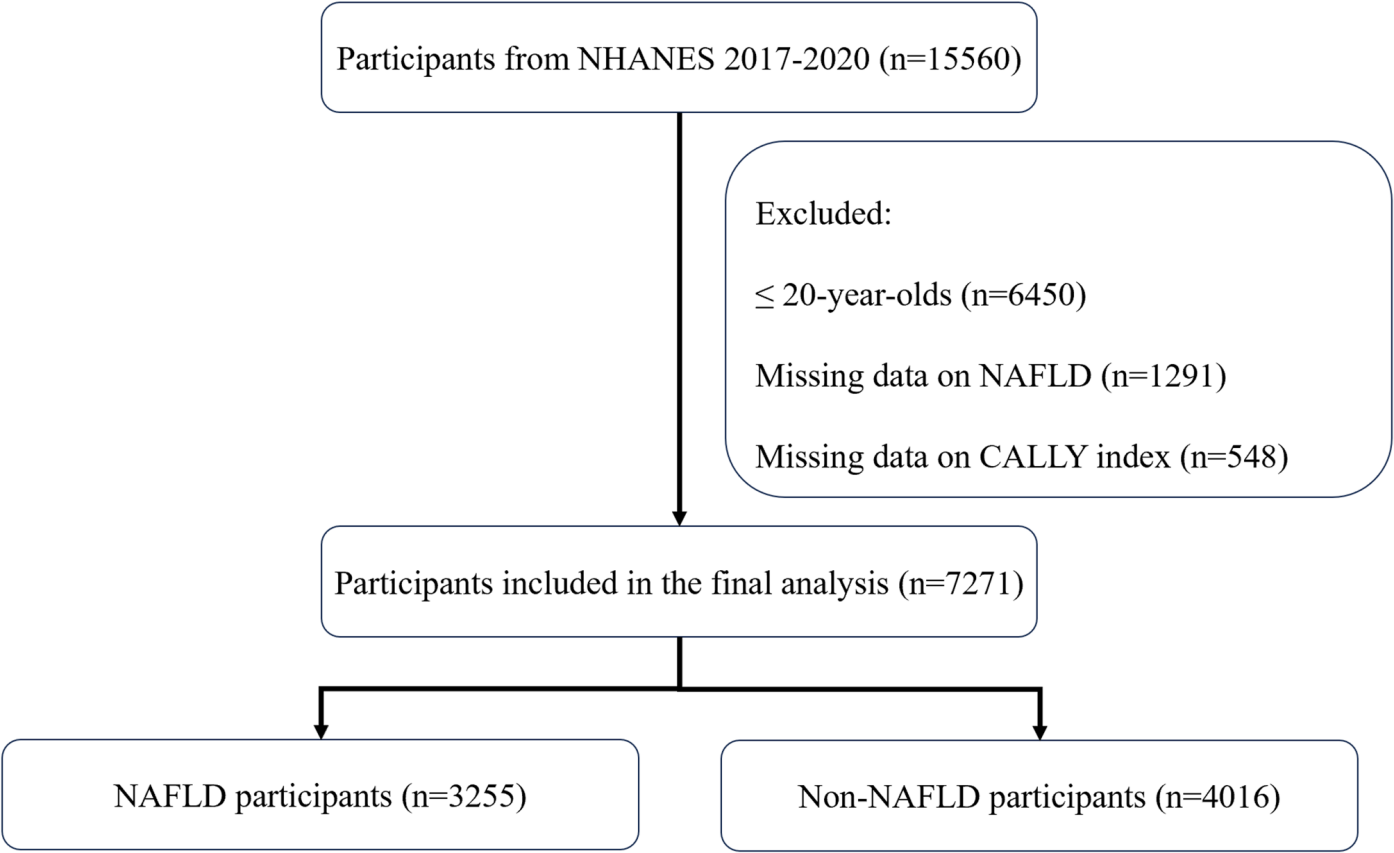
Flowchart of participant selection from NHANES 2017–2020. Flowchart illustrating participant inclusion and exclusion, resulting in 7,271 adults included in the final analysis (3,255 with NAFLD and 4,016 without NAFLD)

The procedure of participant selection is depicted in Fig. 1. This study had 7,271 participants, of which 4,016 were diagnosed with NAFLD and 3,255 were not. The data indicated that, in comparison to non-NAFLD participants, individuals with NAFLD were more frequently older, male, non-Hispanic white, and obese. Furthermore, it is significant that NAFLD patients had much lower CALLY index values than those without the disease, suggesting potential changes in immune function, nutrition, and inflammation. Table 1 delineates the baseline characteristics of all participants.

**Table 1:** Baseline characteristics of the study population

Characteristic	Group	Overall	Non- NAFLD	NAFLD	*P*-value
n		7271	4016	3255	
Age (%)	> 50	3815 (52.5)	1912 (47.6)	1903 (58.5)	< 0.001
	20–50	3456 (47.5)	2104 (52.4)	1352 (41.5)	
Sex (%)	Female	3697 (50.8)	2219 (55.3)	1478 (45.4)	< 0.001
	Male	3574 (49.2)	1797 (44.7)	1777 (54.6)	
Race (%)	Mexican American	886 (12.2)	359 (8.9)	527 (16.2)	< 0.001
	Non-Hispanic black	1844 (25.4)	1162 (28.9)	682 (21.0)	
	Non-Hispanic white	2536 (34.9)	1352 (33.7)	1184 (36.4)	
	Others	2005 (27.6)	1143 (28.5)	862 (26.5)	
BMI (%)	Normal	1707 (23.7)	1480 (37.1)	227 (7.0)	< 0.001
	Obese	5413 (75.1)	2421 (60.8)	2992 (92.9)	
	Underweight	87 (1.2)	84 (2.1)	3 (0.1)	
Education level (%)	Under high school	1348 (18.5)	711 (17.7)	637 (19.6)	0.039
	High school	1731 (23.8)	948 (23.6)	783 (24.1)	
	Above high school	4184 (57.5)	2355 (58.6)	1829 (56.2)	
	No record	8 (0.1)	2 (0.0)	6 (0.2)	
Smoke (%)	No	4198 (57.7)	2360 (58.8)	1838 (56.5)	0.129
	Yes	3070 (42.2)	1654 (41.2)	1416 (43.5)	
	No record	3 (0.0)	2 (0.0)	1 (0.0)	
Activity status (%)	Active	4157 (57.2)	2404 (59.9)	1753 (53.9)	< 0.001
	Inactive	3114 (42.8)	1612 (40.1)	1502 (46.1)	
Drink (%)	No	601 (8.7)	339 (9.0)	262 (8.4)	0.448
	Yes	6296 (91.3)	3444 (91.0)	2852 (91.6)	
Diabetes (%)	No	5949 (81.8)	3550 (88.4)	2399 (73.7)	< 0.001
	Yes	1095 (15.1)	369 (9.2)	726 (22.3)	
	No record	227 (3.1)	97 (2.4)	130 (4.0)	
CAD (%)	No	6953 (95.6)	3875 (96.5)	3078 (94.6)	< 0.001
	Yes	296 (4.1)	127 (3.2)	169 (5.2)	
	No record	22 (0.3)	14 (0.3)	8 (0.2)	
Tumor (%)	No	6538 (89.9)	3645 (90.8)	2893 (88.9)	0.018
	Yes	729 (10.0)	368 (9.2)	361 (11.1)	
	No record	4 (0.1)	3 (0.1)	1 (0.0)	
CAP (mean (SD)) (dB/m)		266.53 (62.54)	220.54 (36.29)	323.27 (35.81)	< 0.001
Albumin (mean (SD)) (g/dL)		40.60 (3.31)	40.80 (3.32)	40.34 (3.29)	< 0.001
Lymphocyte (mean (SD)) (1000 cells/uL)		2.24 (4.34)	2.20 (5.79)	2.30 (0.83)	0.357
CRP (mean (SD)) (mg/L)		4.09 (8.51)	3.32 (8.55)	5.04 (8.37)	< 0.001
CALLY (mean (SD))		8.08 (12.42)	9.99 (15.02)	5.72 (7.49)	< 0.001
CALLY (%)	Q1 (< 1.90)	1818 (25.0)	778 (19.4)	1040 (32.0)	< 0.001
	Q2 (1.90–4.39)	1818 (25.0)	849 (21.1)	969 (29.8)	
	Q3 (4.39-10.00)	1817 (25.0)	1085 (27.0)	732 (22.5)	
	Q4 (> 10.00)	1818 (25.0)	1304 (32.5)	514 (15.8)	

Mean (SD) for continuous variables, % for categorical variables. BMI: body mass index; CAD: chronic kidney disease; CALLY: C-reactive protein-albumin-lymphocyte index; CAP: controlled attenuated parameter; CRP: C-reactive protein

### Association between CALLY index and prevalence of NAFLD

The results of the logistic regression analysis assessing the relationship between the CALLY index and NAFLD are shown in Table 2. In the Model 1, the CALLY index showed a negative association with NAFLD (OR = 0.94, 95% CI = 0.93–0.96), indicating that a higher CALLY index is associated with a lower prevalence of NAFLD. Upon controlling for variables, this negative association persisted as significant (OR = 0.96, 95% CI = 0.95–0.98). Furthermore, when the CALLY index was divided into quartiles, the top quartile exhibited a significantly reduced prevalence of NAFLD in comparison to the lowest quartile (OR = 0.39, 95% CI = 0.24–0.64). These findings endorse the CALLY index’s potential as a serological marker for assessing NAFLD prevalence, rendering it beneficial for early screening and evaluation.

**Table 2:** The relationship between CALLY and NAFLD

		Model 1 OR (95%CI) *P*-value	Model 2 OR (95%CI) *P*-value	Model 3 OR (95%CI) *P*-value
NAFLD	CALLY	0.94 (0.93, 0.96) < 0.001	0.94 (0.92, 0.95) < 0.001	0.96 (0.95, 0.98) 0.002
	Q1	[Reference]	[Reference]	[Reference]
	Q2	0.86 (0.73, 1.03) 0.100	0.80 (0.66, 0.97) 0.028	0.85 (0.65, 1.13) 0.200
	Q3	0.46 (0.36, 0.58) < 0.001	0.41 (0.31, 0.53) < 0.001	0.51 (0.34, 0.76) 0.010
	Q4	0.25 (0.19, 0.34) < 0.001	0.23 (0.17, 0.32) < 0.001	0.39 (0.24, 0.64) 0.006
	P for trend	< 0.001	< 0.001	< 0.001

CALLY: C-reactive protein–albumin–lymphocyte; CI: confidence interval; OR: odds ratio; Q: quartiles

Model 1: no covariates adjusted; Model 2: adjusted for age, sex, and race; Model 3: adjusted for age, sex, race, BMI, educational level, smoke, drink, activity status, diabetes, CAD

### Non-linear association and threshold saturation analysis

**Fig. 2:**
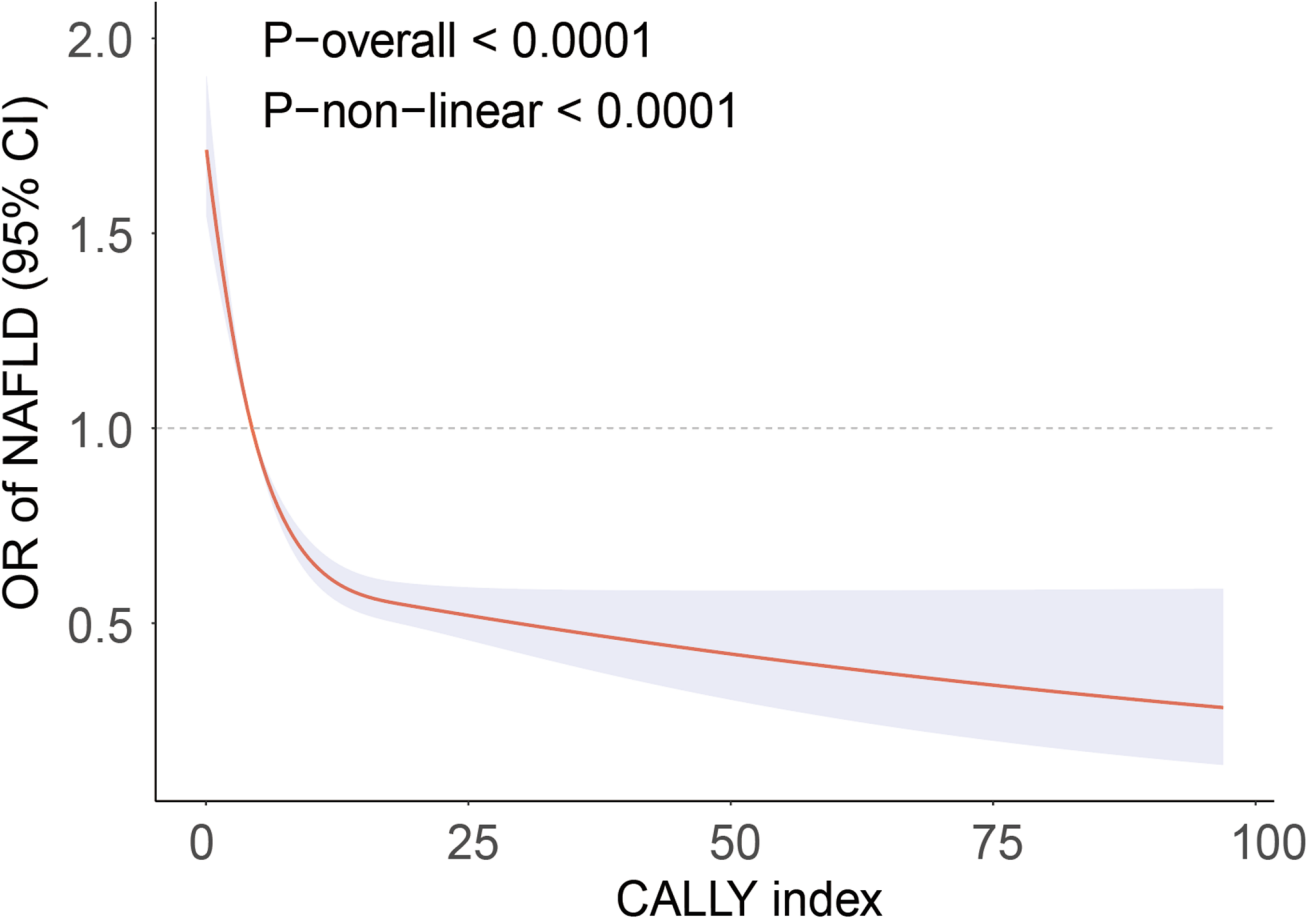
RCS analysis of the association between the CALLY index and NAFLD. Restricted cubic spline showing a significant nonlinear association between the CALLY index and NAFLD prevalence after multivariable adjustment for age, sex, race, BMI, educational level, smoke, drink, activity status, diabetes, CAD

A non-linear inverse association between the CALLY index and NAFLD was shown by RCS analysis (Fig. 2). Subsequent threshold saturation study determined the pivotal inflection point of the CALLY index to be 8.91. The CALLY index was transformed into a binary variable to evaluate its influence on NAFLD. Table 3 presents the findings of the two-segment logistic regression study. To the left of the inflection point (CALLY index < 8.91), each 1-unit increment in the CALLY index corresponded to a 10% reduction in the prevalence of NAFLD (OR = 0.90, 95% CI = 0.88–0.92). Nevertheless, beyond this threshold (CALLY index > 8.91), no significant correlation was detected with the prevalence of NAFLD (*p* > 0.05). These findings indicate that the inverse association between the CALLY index and NAFLD prevalence appears to plateau beyond this threshold.

**Table 3:** Two-stage logistic regression between CALLY and NAFLD

	CALLY	OR (95%CI) *P*-value
NAFLD	Standard linear model	0.97 (0.96, 0.97) < 0.001
	CALLY < 8.91	0.90 (0.88, 0.92) < 0.001
	CALLY > 8.91	0.99 (0.98, 1.00) 0.120
	Log-likelihood ratio test	< 0.001

CALLY: C-reactive protein–albumin–lymphocyte; CI: Confidence Interval; OR: Odds Ratio;

### Subgroup analysis

Taking demographic and lifestyle factors into account, subgroup studies were conducted to examine the impact of various populations on the relationship between the CALLY index and NAFLD. The negative relationship between the CALLY index and NAFLD remained consistent across all categories after adjusting for all variables (Table 4). A notable interaction between the CALLY index and age was identified for NAFLD. The results indicate that the CALLY index could function as a broadly applicable biomarker across diverse populations.

**Table 4:** Subgroup analysis between CALLY and NAFLD

Characteristic	Group	OR (95%CI) *P*-value	*P* for interaction
Age	< 50	0.96 (0.93, 0.98) 0.002	0.013
	> 50	0.97 (0.96, 0.99) 0.007	
Sex	Male	0.97 (0.95, 0.99) 0.010	0.091
	Female	0.95 (0.92, 0.97) 0.002	
Race	Mexican American	0.96 (0.92, 1.00) 0.062	0.120
	Non-Hispanic black	0.97 (0.95, 1.00) 0.026	
	Non-Hispanic white	0.96 (0.94, 0.98) 0.002	
	Others	0.98 (0.97, 0.99) 0.011	
Smoke	No	0.96 (0.94, 0.98) < 0.001	0.800
	Yes	0.97 (0.94, 0.99) 0.013	
Drinking	No	0.99 (0.96, 1.02) 0.500	0.062
	Yes	0.96 (0.95, 0.98) 0.001	
Activity Status	Active	0.96 (0.94, 0.98) 0.004	0.500
	Inactive	0.97 (0.95, 0.99) 0.014	

### Sensitivity analysis

To assess the robustness of the findings, multiple sensitivity analyses were conducted. After excluding participants with CALLY index values beyond the mean ± 3 SD, 7,168 individuals were included, comprising 3,924 without NAFLD and 3,241 with NAFLD. As shown in Supplemental Table 1, the negative association between the CALLY index and NAFLD remained significant across unadjusted, partially adjusted, and fully adjusted models, with consistent trend tests. Further exclusion of individuals with a history of tumor yielded 6,538 participants (3,645 without NAFLD and 2,893 with NAFLD), and the results in Supplemental Table 2 were concordant with the primary analysis. These findings indicate that the inverse association between the CALLY index and NAFLD is robust across different analytical scenarios.

## Discussion

This research examined the correlation between the CALLY index and NAFLD, revealing a substantial inverse link between the CALLY index and the prevalence of NAFLD. A heightened CALLY index correlates with a reduced incidence of NAFLD, offering valuable information for early NAFLD screening. The CALLY index encompasses three indicators: albumin, lymphocyte count, and CRP, collectively reflecting the body’s immunological, nutritional, and inflammatory health. Consequently, the CALLY index may function as a supplementary screening instrument for NAFLD, particularly for patients who cannot undergo liver biopsy. Its simplicity and cost-effectiveness suggest potential value as an indicator for further research on clinical screening approaches, which may inform future studies aimed at early intervention.

This study systematically evaluated the CALLY index as a comprehensive biomarker for NAFLD, comparing it to traditional approaches that rely on single biomarkers such as albumin, lymphocyte count, or CRP [18, 37, 38]. While these individual markers offer early warnings, they often fail to capture the full complexity of NAFLD pathophysiology. In addition, commonly used noninvasive indices, such as the NAFLD Fibrosis Score, FIB-4, and APRI, primarily assess hepatic fibrosis rather than earlystage steatosis and do not incorporate systemic inflammatory or nutritional components. In contrast, the CALLY index integrates inflammatory and nutritional markers, potentially providing complementary information for identifying early metabolic disturbances in NAFLD. The study found that a higher CALLY index was linked to a lower prevalence of NAFLD, supporting theories about the beneficial role of immune function and nutritional status in slowing hepatic fat accumulation. This suggests that the CALLY index may be associated with better immune function, reduced inflammation, and improved nutritional status, all of which help slow liver fat accumulation [39–42]. However, once the CALLY index reaches a critical threshold, further increases are not significantly associated with NAFLD prevalence, suggesting that the inverse association plateaus beyond this point. These findings suggest that although the CALLY index shows an inverse association with NAFLD prevalence, this association appears stronger in the earlier stages of disease and may weaken in later stages. Subgroup analysis showed that the CALLY index maintained a negative association with NAFLD across different subgroups, indicating its broad applicability as a predictive biomarker in various populations. However, the interaction between age and the CALLY index revealed that the negative association with NAFLD was stronger in younger populations and weaker in older individuals, which may be related to age-associated changes in immune function and metabolic status. Given its simplicity and low cost, the CALLY index may represent a potential non-invasive indicator associated with NAFLD, particularly useful for early screening in high-risk populations who are unable to undergo invasive tests. Importantly, although the magnitude of the association per unit change in the CALLY index may appear modest, this consistent inverse trend could still hold meaningful implications for population-level disease burden and clinical risk stratification, especially in the context of NAFLD’s high prevalence. Nonetheless, the clinical applicability of this finding requires validation through prospective studies to confirm its prognostic value and effectiveness in monitoring disease progression.

The negative association between the CALLY index and NAFLD may be linked to its reflection of immune, nutritional, and inflammatory status. Albumin, a key indicator of nutritional health, possesses antioxidant and anti-inflammatory properties [43, 44]. Decreased albumin levels are commonly observed in chronic inflammation and malnutrition, with low levels in liver damage potentially leading to immune dysfunction and worsening fatty liver progression [45]. Lymphocyte count, an indicator of immune function, is often reduced in cases of immune suppression and chronic inflammation. A decrease in lymphocytes can impair immune response, leading to fat accumulation in the liver and steatosis [46, 47]. Moreover, CRP, a marker of systemic inflammation, is generally elevated in conditions like chronic low-grade inflammation, fatty liver, and liver damage [48, 49]. Elevated CRP exacerbates oxidative stress and inflammatory response, speeding up NAFLD progression [50, 51]. A higher CALLY index typically reflects lower levels of inflammation, improved immune function, and better nutritional status, which are associated with a lower likelihood of liver fat accumulation and a reduced prevalence of NAFLD. These findings suggest that the CALLY index provides a new tool for early NAFLD screening and may offer a biological foundation for clinical interventions.

This study’s strengths lie in the novel introduction and validation of the CALLY index as a potential biomarker for NAFLD. Utilizing the large, nationally representative NHANES database, the study ensured high statistical power and broad external validity. Additionally, by controlling for potential confounding factors through multivariate analysis, the reliability of the conclusions was enhanced. However, as a cross-sectional study, it only identifies the association between the CALLY index and NAFLD, without establishing causal relationships. While the NHANES data is representative, it is predominantly from the U.S., and the sample’s racial and regional characteristics may limit the generalizability of the findings, particularly in other countries or ethnic groups. In addition, NAFLD was defined using hepatic steatosis measured by VCTE; although widely used in epidemiological studies, this method cannot differentiate simple steatosis from NASH, which may limit interpretation of disease heterogeneity. Moreover, VCTE measurements may be affected by interoperator variability and body composition, potentially introducing measurement bias. Despite adjusting for multiple covariates, residual confounding from unmeasured factors such as medication use or genetic predisposition cannot be entirely excluded. Furthermore, the study relied on static data and did not account for the impact of temporal changes in the CALLY index on NAFLD. Future longitudinal studies are necessary to confirm the CALLY index’s predictive ability in NAFLD progression.

## Conclusion

The CALLY index demonstrates a significant inverse association with NAFLD prevalence, suggesting its potential utility as a comprehensive biomarker for identifying individuals at higher likelihood of NAFLD and providing insights that may inform future research on prevention and intervention strategies.

## Supplementary Information

Below is the link to the electronic supplementary material.

Supplementary Material 1

## Data Availability

The NHANES statistics are valuable for scholars and data users globally. All data is accessible at https://wwwn.cdc.gov/nchs/nhanes/default.aspx.
