# Supplementary material for: Association of CALLY index with NAFLD in U.S. adults from NHANES 2017–2020 assessed by vibration-controlled transient elastography
Source: Diabetol Metab Syndr. 2025 Aug 29;17:363. doi: 10.1186/s13098-025-01926-y (PMC12395825; doi:10.1186/s13098-025-01926-y)
Supplement: Supplementary file 1 — Supplementary Material 1 [file 13098_2025_1926_MOESM1_ESM.docx]

**Supplemental Table 1 Sensitivity Analysis of the Association Between CALLY Index and NAFLD After Excluding Extreme CALLY Values.**

|  |  | Model 1  OR (95%CI) P-value | Model 2  OR (95%CI) P-value | Model 3  OR (95%CI) P-value |
| --- | --- | --- | --- | --- |
| NAFLD | CALLY | 0.93 (0.92, 0.95) <0.001 | 0.93 (0.92, 0.95) <0.001 | 0.96 (0.94, 0.98) 0.001 |
|  | Q1 | [Reference] | [Reference] | [Reference] |
|  | Q2 | 0.86 (0.73, 1.03) 0.100 | 0.80 (0.66, 0.97) 0.028 | 0.85 (0.65, 1.12) 0.200 |
|  | Q3 | 0.46 (0.36, 0.58) <0.001 | 0.41 (0.31, 0.53) <0.001 | 0.50 (0.34, 0.76) 0.010 |
|  | Q4 | 0.26 (0.20, 0.35) <0.001 | 0.24 (0.17, 0.33) <0.001 | 0.40 (0.25, 0.65) 0.006 |
|  | P for trend | <0.001 | <0.001 | 0.001 |

CALLY: C-reactive protein–albumin–lymphocyte; CI: confidence interval; OR: odds ratio; Q: quartiles

Model 1: no covariates adjusted; Model 2: adjusted for age, sex, and race; Model 3: adjusted for age, sex, race, BMI, educational level, smoke, drink, activity status, diabetes, CAD.

**Sensitivity Analysis of the Association Between CALLY Index and NAFLD After Excluding Participants With Tumor.**

|  |  | Model 1  OR (95%CI) P-value | Model 2  OR (95%CI) P-value | Model 3  OR (95%CI) P-value |
| --- | --- | --- | --- | --- |
| NAFLD | CALLY | 0.94 (0.93, 0.96) <0.001 | 0.94 (0.92, 0.96) <0.001 | 0.96 (0.95, 0.98) 0.003 |
|  | Q1 | [Reference] | [Reference] | [Reference] |
|  | Q2 | 0.88 (0.72, 1.06) 0.200 | 0.81 (0.66, 1.00) 0.054 | 0.88 (0.65, 1.18) 0.300 |
|  | Q3 | 0.45 (0.36, 0.57) <0.001 | 0.40 (0.31, 0.53) <0.001 | 0.50 (0.33, 0.75) 0.009 |
|  | Q4 | 0.26 (0.19, 0.35) <0.001 | 0.23 (0.17, 0.33) <0.001 | 0.40 (0.24, 0.65) 0.007 |
|  | P for trend | <0.001 | <0.001 | 0.001 |

CALLY: C-reactive protein–albumin–lymphocyte; CI: confidence interval; OR: odds ratio; Q: quartiles

Model 1: no covariates adjusted; Model 2: adjusted for age, sex, and race; Model 3: adjusted for age, sex, race, BMI, educational level, smoke, drink, activity status, diabetes, CAD.
